# Metformin-Associated Encephalopathy as a Rare but Serious Adverse Effect: A Case Report

**DOI:** 10.7759/cureus.101758

**Published:** 2026-01-17

**Authors:** Anna Aoki, Daisuke Mizu, Hidenori Higashi, Jumpei Yamashita

**Affiliations:** 1 Department of Emergency Medicine, Japanese Red Cross Osaka Hospital, Osaka, JPN

**Keywords:** adverse effect, diabetic nephropathy, lentiform fork sign, metformin, mri, parkinsonism

## Abstract

Metformin is widely prescribed as a first-line therapy for type 2 diabetes. Metformin-associated encephalopathy is a rare but serious adverse effect that can occur when the drug is inappropriately prescribed to patients with impaired renal function. Here we present the case of a 44-year-old woman undergoing maintenance hemodialysis for diabetic nephropathy who presented to the emergency department with dysarthria and generalized weakness. Although no paralysis was observed, she had difficulty maintaining posture and exhibited gait disturbance. Laboratory tests revealed metabolic acidosis (pH 7.197, bicarbonate 11.7 mmol/L) and hyperlactatemia (12.4 mmol/L). Brain magnetic resonance imaging (MRI) revealed hyperintensity in the bilateral basal ganglia. The patient had been taking metformin. Metformin was discontinued, and emergent hemodialysis was initiated, followed by continued maintenance hemodialysis; the patient’s dysarthria, weakness, and abnormal findings on brain MRI subsequently rapidly improved, which confirmed the diagnosis of metformin-associated encephalopathy. Inappropriate prescription of metformin can lead to metformin-associated encephalopathy, a rare but serious adverse effect. Emergency physicians must carefully review of patients’ medications and become familiar with the clinical and imaging features of metformin-associated encephalopathy.

## Introduction

Metformin is widely prescribed for people with type 2 diabetes, as it rarely causes hypoglycemia and is generally considered a safe medication. However, it is nevertheless associated with some adverse effects, with gastrointestinal symptoms such as diarrhoea, vomiting, and abdominal pain being the most commonly reported [1-3]. Metformin-associated lactic acidosis (MALA) is recognized as a serious adverse effect, although its incidence is approximately 10 cases per 100,000 patients [4]. While metformin is contraindicated in patients with severe renal impairment [5], in practice, it is still prescribed to approximately 2-10% of patients with renal impairment [6].

Emergency physicians must be able to accurately identify conditions caused by adverse effects of medications, as failure to discontinue the medication or initiate appropriate treatment may result in poorer outcomes. Metformin-associated encephalopathy is a rare adverse effect compared with MALA and remains insufficiently recognized among emergency physicians. Its nonspecific clinical symptoms necessitate a broad differential diagnosis, while characteristic findings on brain magnetic resonance imaging (MRI) have been described [3,7-9]. To aid in the appropriate evaluation of metformin-associated encephalopathy, we report a case showing the typical clinical presentation and imaging characteristics of this condition.

## Case presentation

The patient, a 44-year-old woman, was transported to the emergency department due to dysarthria and generalized weakness, which had been present for the past two days. She had a past medical history of type 2 diabetes mellitus, hypothyroidism, insomnia, and hypertension. She had been receiving maintenance hemodialysis three times per week at her primary care clinic due to diabetic nephropathy. The patient was slightly somnolent but able to communicate. Her vital signs were as follows: blood pressure of 214/139 mmHg, heart rate of 100 beats/min, respiratory rate of 24 breaths/min, oxygen saturation level of 100% (in room air), and body temperature of 37°C. The pupils were 3 mm bilaterally, with prompt and complete light reflex, while ocular movement was normal. She showed no motor paralysis or sensory disturbance in the extremities; however, she had difficulty maintaining sitting and standing positions and exhibited dysarthria. No other abnormal physical findings were noted. Blood tests revealed metabolic acidosis (pH 7.19, bicarbonate 11 mmol/L), hyperlactatemia (12.4 mmol/L), and hyperkalemia (6.2 mEq/L) (Table 1).

**Table 1:** Laboratory data in the emergency department. CBC: complete blood count; TB: total bilirubin; AST: aspartic aminotransferase; ALT: alanine aminotransferase; LDH: lactate dehydrogenase; BUN: blood urea nitrogen; Cr: creatinine; eGFR: estimated glomerular filtration; Na: sodium; K: potassium; Glu: glucose; CRP: C-reactive protein; WBC: white blood cell; RBC: red blood cell; Hb: hemoglobin; Hct: hematocrit; Pl: platelet; HCO3-: bicarbonate; BE: base excess.

Biochemistry	Value	Normal range
TB (mg/dL)	0.2	0.4-1.5
AST (IU/L)	30	13-30
ALT (IU/L)	16	23-Jul
LDH (IU/L)	501	124-222
BUN (mg/dL)	54.8	20-Aug
Cr (mg/dL)	7.4	0.46-0.79
eGFR (ml/min/1.73m2)	5.4	≥60
Na (mEq/L)	135	138-145
K (mEq/L)	6.2	3.6-4.8
Glu (mg/dL)	124	70-110
CRP (mg/dL)	0.49	0-0.5
CBC		
WBC (×103/μL)	7.44	3.3-8.6
RBC (×104/μL)	313	350-510
Hb (g/dL)	10.5	11.6-14.8
Hct (%)	33.6	35.1-44.4
Plt (×104/μL)	13.2	15.8-34.8
Acid–base/blood gas balance		
pH	7.19	7.35-7.45
pCO2 (mmHg)	29.1	35-45
HCO3-, mmol/L	11	20-26
BE, mmol/L	-15.7	-6
Lactate, mmol/L	12.4	0.5-2

**Figure 1:**
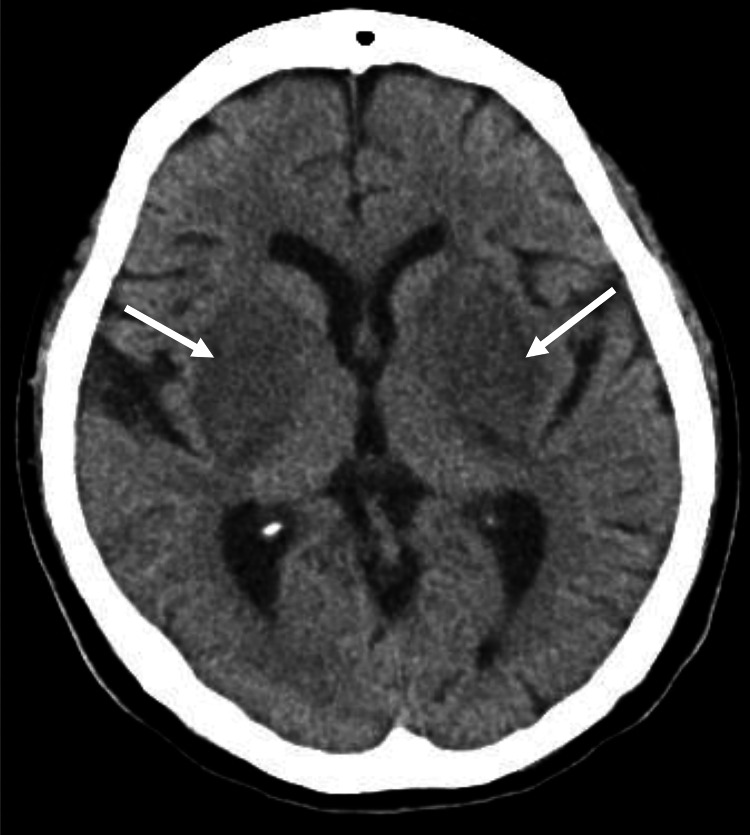
Brain computed tomography performed in the emergency department Low density area were observed in the bilateral basal ganglia (arrows)

**Figure 2:**
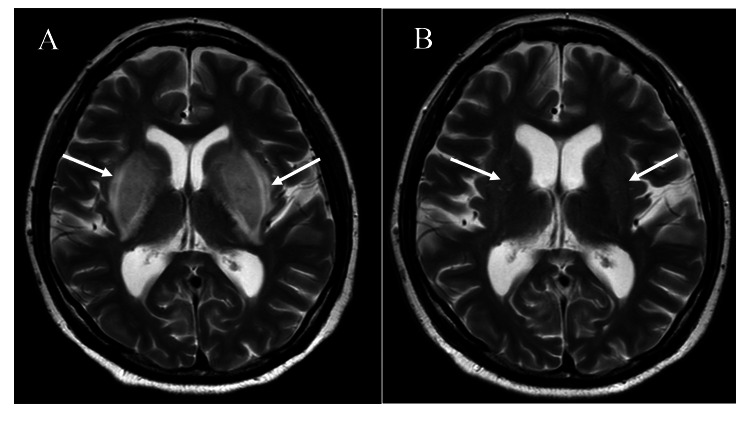
T2-weighted brain MRI performed in the emergency department (A) and follow-up brain MRI performed on hospital day 14 (B) A: Hyperintensity lesions were observed in the bilateral basal ganglia, presenting as the “Lentiform fork sign” B: The previously noted “Lentiform fork sign” had disappeared (arrows).

Brain computed tomography (CT) revealed hypodensity in the bilateral basal ganglia (Figure 1), while brain MRI revealed hyperintensity in the bilateral basal ganglia on T2-weighted and fluid attenuated inversion recovery (FLAIR) images, consistent with the characteristic finding known as the “Lentiform fork sign” (Figure 2A). Assessment of her medication records revealed that the patient had been prescribed immediate-release metformin 1000mg/day at the primary care clinic starting one month prior.

Metformin was discontinued, and emergent hemodialysis was initiated. Subsequently, maintenance hemodialysis was conducted three times per week, leading to a rapid improvement of dysarthria. A follow-up brain MRI performed on hospital day 14 confirmed the disappearance of the lentiform fork sign (Figure 2B). For hypertension, a continuous intravenous infusion of nicardipine hydrochloride was initiated in the emergency department, resulting in a rapid reduction of systolic blood pressure to approximately 180 mmHg. On hospital day 2, blood pressure control was transitioned to oral amlodipine besilate at a dose of 5 mg/day. From hospital day 4 onward, systolic blood pressure was maintained between 130 and 150 mmHg. Because MRI showed no lesions in the parietal or occipital lobes and demonstrated a lentiform fork sign, the findings were not typical of hypertensive encephalopathy.

In addition, considering the patient’s medication history and the presence of lactic acidosis, the patient was diagnosed with metformin-associated lactic acidosis and metformin-associated encephalopathy. Dysarthria resolved by hospital day 5; however, gait disturbance improved only partially and persisted, with the patient able to walk slowly with residual unsteadiness. Because the patient lived alone, lacked sufficient family support, and had significant anxiety about returning to daily life, we decided to transfer to a hospital that could provide rehabilitation-focused treatment for gait disturbance. Transfer arrangements were initiated on hospital day 15; however, coordination was challenging, and the transfer was ultimately completed on hospital day 35. 

## Discussion

Among the various adverse effects of metformin, metformin-associated lactic acidosis (MALA) is the most widely recognized; however, metformin-associated encephalopathy is scarcely recognized [1]. This case is a typical and important report of metformin-associated encephalopathy, demonstrating that metformin-related adverse effects may involve not only lactic acidosis but also neurological symptoms such as gait disturbance and dysarthria, as well as abnormal findings in the bilateral basal ganglia on brain MRI. However, as discussed below, there are dialysis-related conditions with pathophysiological features that overlap with those of metformin-associated encephalopathy, and it should be recognized that a clear distinction may sometimes be difficult.

Metformin is excreted by the kidneys, meaning that caution is required when prescribing it to patients with renal impairment. The U.S. Food and Drug Administration (FDA) contraindicates its use in patients with an estimated glomerular filtration rate (eGFR) <30 mL/min/1.73 m², and does not recommend its use in those with an eGFR of 30-40 mL/min/1.73 m² [5]. However, some reports indicated that the risk of developing MALA in patients with an eGFR of 30-40 mL/min/1.73 m² is low, indicating that metformin use may be relatively safe [10]. In practice, metformin is prescribed to 10.6% of patients with an eGFR of 15-29 mL/min/1.73 m² and to 2.1% of those with an eGFR <15 mL/min/1.73 m² [6]. As such, the use of metformin in patients with renal impairment is not negligible and must always be carefully assessed.

The mechanisms by which metformin leads to lactate accumulation are multifactorial. Metformin acts by inhibiting the mitochondrial complex I of the respiratory chain to inhibit adenosine triphosphate (ATP) production, leading to decreased gluconeogenesis and increased anaerobic metabolism. In addition, Metformin inhibits hepatic gluconeogenesis and the conversion of lactate to pyruvate, resulting in reduced lactate metabolism [2,4,8]. The mitochondria in the basal ganglia are particularly vulnerable to metformin, and localized lactate accumulation may lead to the development of neurological symptoms [8,9]. 

The effect on the basal ganglia may be recognized on MRI as the “Lentiform fork sign”, a characteristic MRI finding described as bilateral symmetric hyperintensity in the basal ganglia, accompanied by surrounding hyperintensity in the adjacent white matter [7-9,11-13]. However, the sign has been reported in various conditions, including severe metabolic acidosis, intoxication, hypoglycemia, and hypoxia, and is not specific to any particular disease [8,11-13]. The lentiform fork sign has further been suggested to result from vasogenic edema associated with metabolic acidosis, although its mechanism has not been fully understood [14]. Indeed, lentiform fork signs have been reported even in metformin-associated encephalopathy without metabolic acidosis [15]. As mentioned earlier, lactate accumulation in the basal ganglia is also considered a contributing factor; therefore, metformin may be regarded as a risk factor.

There are currently no established diagnostic criteria for metformin-associated encephalopathy; the diagnosis is made clinically, based on the rapid improvement of symptoms and MRI findings following cessation of metformin and supportive therapy [7,16]. Further, measurement of serum metformin levels is not necessarily required to establish the diagnosis of metformin-associated encephalopathy [3]. In this case, all symptoms except for gait disturbance improved rapidly after discontinuation of metformin and initiation of dialysis, and the abnormal MRI findings resolved by day 14. This clinical course and the imaging findings are consistent with a diagnosis of metformin-associated encephalopathy when compared with previous reports [3,8,13,16,17].

In the emergency department, the patient had marked hypertension, and hypertensive encephalopathy needed to be considered in the differential diagnosis. Brain MRI is useful for the diagnosis of hypertensive encephalopathy, and white matter lesions in the parieto-occipital lobes are commonly observed [18]. Although isolated abnormalities in the basal ganglia have been reported [18], there are no reports describing a lentiform fork sign as observed in this case. Therefore, based on the MRI findings, this case was not typical of hypertensive encephalopathy, and a diagnosis of metformin-associated encephalopathy was considered more appropriate. Nevertheless, because the lentiform fork sign may involve vascular edema, a contribution from hypertension cannot be completely excluded. 

There are several conditions which are clinically similar to metformin-associated encephalopathy. Wang et al. [19] proposed diabetic uremic syndrome as a condition characterized by acute-onset motor disturbances and bilateral basal ganglia lesions in patients with diabetic nephropathy undergoing maintenance dialysis. In addition, Manickavasagar et al. [20] proposed extrapyramidal syndromes of chronic kidney disease and dialysis (EPS-CKDD) to describe a condition in which patients with chronic diabetes on maintenance dialysis develop acute neurological symptoms, typically Parkinsonism, accompanied by abnormal findings in the bilateral basal ganglia on brain MRI. It is often difficult to clinically differentiate between diabetic uremic syndrome, EPS-CKDD, and metformin-associated encephalopathy, as these conditions may overlap [17]. However, metformin is thought to contribute to the development of encephalopathy. Further studies are warranted to clarify the distinctions and possible integration among these conditions.

## Conclusions

Metformin-associated encephalopathy is a rare but serious adverse effect. Because there are no specific symptoms or imaging findings, emergency physicians should carefully review patients’ medication histories and be familiar with the characteristic clinical and imaging features of metformin-associated encephalopathy to facilitate early diagnosis and treatment.
